# Accessibility of Special Care Dentistry Across Countries: A Scoping Review

**DOI:** 10.3390/healthcare12232376

**Published:** 2024-11-26

**Authors:** Amin Vahdati, Gita Khadivi, Zahra Ghorbani, Ehsan Vahdati Helan, Anahita Ranjbar, Somayyeh Azimi

**Affiliations:** 1 Department of Community Oral Health, Dental School, Shahid Beheshti University of Medical Sciences, Tehran 1983969411, Iran; 2 Centre for Biostatistics, Division of Population Health, Health Services Research & Primary Care, The University of Manchester, Manchester M13 9PL, UK; 3 USERN Office, Shahid Beheshti University of Medical Sciences, Tehran 1983969411, Iran; 4 Department of Internal Medicine, Faculty of Medicine, Tabriz University of Medical Science, Tabriz 9183795189, Iran; 5 Department of Community Oral Health, School of Dentistry, Kashan University of Medical Sciences, Esfahan 8715981151, Iran; 6 School of Allied Health, University of Western Australia, Crawley, WA 6009, Australia

**Keywords:** patients with special needs, special care dentistry, oral health, dental care

## Abstract

Introduction: People with special care needs often face significant barriers in accessing dental care due to physical and cognitive limitations, leading to high rates of dental issues like caries. Despite the growing recognition of these challenges, unmet dental care needs remain prevalent. This review aims to explore the global landscape of special care dentistry to identify gaps and opportunities for improving dental services for this population. Methodology: A systematic search was conducted across three online databases—PubMed, Embase, and Scopus—to identify relevant articles from their inception through May 2024. Reference lists of the selected studies were also screened for additional sources. A thematic synthesis approach was applied to derive both descriptive and analytical themes. The scoping review was guided by the Arksey and O’Malley framework to examine the scope and nature of studies and documents related to dental care for individuals with special care needs. Furthermore, a Google search was performed to include accessible theses and official government documents from various countries. Results: A total of 49 studies from 11 countries were reviewed, all centered on providing dental care for people with special needs. The analysis revealed three main themes: Human resources, care delivery model, and management. Within these, nine subthemes emerged: Mid-level oral care providers, dentists, special care dentistry as a specialty, tele-dentistry, mobile dentistry, hospital care, levels of healthcare provision, financial support, and education. These themes and subthemes highlight essential areas for enhancing services for those people. Conclusions: A holistic approach is essential to enhance dental care for people with special needs. Critical strategies, including the involvement of mid-level oral care providers, the adoption of tele-dentistry and mobile units, and the availability of hospital-based services for complex cases, are crucial. To truly transform care for those people, each country must adapt these strategies to its specific context, aligning resources and policies to create an inclusive, accessible, and effective system.

## 1. Introduction

Accessibility of healthcare services, particularly dental care, is an essential need for patients with special needs (PWSNs). In this context, PWSNs are defined as individuals facing physical, sensory, intellectual, mental, medical, emotional, or social challenges, often involving a combination of these factors [1]. Due to these physical and cognitive limitations, this group often relies more heavily on oral health care, as these challenges can significantly impede their ability to maintain proper oral hygiene [2,3]. As a result, they frequently encounter various dental health issues in adulthood, with dental caries being particularly prevalent in this population [4,5]. The most recent study on dental caries prevalence found that caries affected 25% to 50% of the general population’s permanent teeth, with significantly higher rates of 89.4% to 95.5% among individuals with intellectual disabilities [6]. Studies focused on assessing the dental health status of PWSNs consistently reveal poorer oral hygiene and periodontal health, a higher prevalence of untreated caries, and fewer remaining teeth [4,7]. It becomes evident that this group often encounters unmet dental needs, indicating that the current utilization of dental services is insufficient in adequately meeting their requirements [8,9].

Patients with special needs often face substantial barriers to accessing dental care, primarily due to inadequate healthcare policies, limited socioeconomic resources, a shortage of trained dental professionals, and a lack of accessible healthcare facilities [10]. The report highlights significant obstacles across these diverse healthcare systems, citing cost as a barrier for up to 65.8% in the USA, communication challenges for 47.4% in Canada, and physical accessibility issues for up to 64.3% in the UK as major barriers to dental care for special needs patients in the USA, UK, and Canada. Health professionals and carers report that these systemic issues create significant gaps in the infrastructure and workforce, making it challenging to meet the unique dental needs of this population [11]. A national survey in the United States underscores this issue, revealing that while 78% of children with special healthcare needs required dental services in the past year, 41% were unable to obtain the care they needed [12].

As the number of PWSNs who require specialized healthcare services keeps increasing [13,14], there is a growing need to address their oral health. Historically, PWSNs have been overlooked in social planning and policy making, and fragmented healthcare services have further disadvantaged this vulnerable group [15]. These challenges are exacerbated in low- and middle-income countries, where public health infrastructure often struggles to support the specialized requirements of this population [12].

Despite the evident challenges faced by PWSNs in accessing oral care, there remains a significant gap in the literature regarding comprehensive assessments of existing resources and practices in Special Care Dentistry (SCD) globally. While various studies highlight the disparities in dental health outcomes among PWSNs [16,17], they often fail to provide an integrated perspective on how different countries are addressing these challenges.

To address this gap, the current study aims to investigate the global landscape of SCD with an emphasis on understanding how countries with varying healthcare systems and economic conditions address the dental needs of PWSNs. This review will not only assess existing policies and resources but also highlight the specific limitations within these systems that hinder equitable access to care. It aims to contribute to the development of public health strategies that prioritise accessible and inclusive dental care, ultimately advancing oral health equity on a global scale.

## 2. Method

Our approach follows Arksey and O’Malley’s methodological framework for scoping reviews, incorporating a modified version of the recommended five-step process [18]. We adhered to the PRISMA-ScR guidelines to ensure transparency and completeness in reporting our systematic review [19].

### 2.1. Identification of Research Questions and Relevant Research Studies

The scoping review aimed to answer the main question of how various countries provide dental care services for PWSNs and what approaches they employ. To maximize comprehensiveness, we applied a diverse array of keywords, encompassing terms such as “oral health”, “oral health care”, “dentistry”, “dental care”, “dental service”, and “Special Care Dentistry”. Additionally, we coupled the selected keywords with those of the concept of disability, including “disab*”, “disorder”, “mental retard”, “deficiency”, and “special needs”. (Table S1: Search strategy in PubMed).

We implemented a meticulous search strategy to identify pertinent studies in this scoping review. We utilized a range of electronic resources, including PubMed, Scopus, and Embase, to conduct our search from 2006 to 12 October 2024. We also used the Google search engine to locate additional relevant references cited within the articles included in our review. This approach helped ensure comprehensive coverage by identifying studies that may not have been indexed in the primary databases.

We designed and deliberated collaboratively on this strategy to optimize the scope and relevance of this review. Subsequently, we removed duplicates, matched obtained papers against eligibility criteria, and extracted data from the included papers during the search process. Two of the authors involved in the study scrutinized the results and interpreted the data collaboratively.

### 2.2. Study Selection

The scoping review applied specific eligibility criteria to ensure the inclusion of relevant studies. Studies were required to (1) address individuals with special care needs or those requiring special care dentistry, (2) describe or reference the methods used to deliver healthcare services to these individuals within the healthcare system, and (3) be published in English. Studies that focused on dentist–patient interactions, patient satisfaction with access to services, or the prevalence of oral and dental diseases in this demographic were excluded. Additionally, studies centered on the general population without specific emphasis on PWSNs, those lacking detailed descriptions of healthcare delivery methods, and those that focused solely on patient-centered perspectives were not included. Publications in languages other than English and duplicate studies were also excluded.

### 2.3. Charting the Data

Two researchers independently conducted a two-stage screening process. In the first stage, they reviewed titles and abstracts to determine adherence to the inclusion criteria. For articles where a definitive decision could not be made based on the title and abstract alone, the introduction of the article was also examined. Articles were then categorized as “related”, “potentially related”, or “unrelated”. In the second stage of screening, articles categorized as potentially related were subjected to a final eligibility assessment. Face-to-face meetings were held to reach a consensus on articles where disagreements arose.

### 2.4. Collating, Summarizing, and Reporting Results

We employed a content analysis method based on Graneheim and Lundman’s qualitative approach to extract actionable recommendations from the data [20]. Graneheim and Lundman’s qualitative content analysis systematically codes and categorizes textual data to identify themes and patterns. This approach is beneficial in scoping reviews, synthesizing diverse qualitative studies to enhance understanding of complex phenomena, ensuring rigor and clarity in interpreting findings, and guiding future research directions. AV and GK conducted a two-stage screening process to review and select relevant studies, assessing titles, abstracts, and introductions in the first stage and independently reviewing potentially relevant articles in the second stage. We used EndNote X20.1 software and held face-to-face meetings for consensus. The process involved a comprehensive reading of all articles, followed by analysis and coding of words, sentences, and paragraphs as meaningful units. These units were abstracted to conceptual levels, coded, compared, and categorized into main groups. We considered the entire text of the articles as the unit of analysis. We identified units of meaning, coded them, and organized them into subcategories and main categories. We further categorized latent content within the data into subthemes and main themes.

## 3. Results

In total, 6006 articles were extracted. Of these, 3449 articles were obtained from the PubMed database, 1910 from Scopus, and 647 from the Embase database. After removing duplicate articles, 482 articles were eliminated, leaving 5622 articles. In the initial screening phase, the titles and abstracts of these articles were examined. Two independent reviewers (A.V. and G.K.) also assessed the introductory sections of potentially relevant articles. Subsequently, 5240 articles were removed during this stage, leaving 382 articles. Based on inclusion and exclusion criteria, 344 articles were removed. During this phase, websites related to certain articles were searched, and five websites were also examined for data extraction. Consequently, a total of 43 sources were incorporated for the final review and data extraction. The article selection process is visualized in Figure S1. The number of articles categorized by country is detailed in Table S2.

### 3.1. Analytical Framework and Themes in SCD

Each article’s entire text was considered an analytical unit in this phase. It was thoroughly studied to comprehensively understand its content. Finally, the content within the data was formulated into three main themes and nine subthemes. The three main themes extracted as primary themes are “Human Resources”, “Care Delivery Model”, and “Management”. The main themes and subthemes for providing strategies for services to PWSNs are summarized in Table 1.

### 3.2. Human Resource

The theme of “Human Resources” emerged as a critical factor in the delivery of dental care services to PWSNs across different countries. Within this theme, three subthemes were identified: Mid-level oral care providers, Dentists, and SCD.

#### 3.2.1. Mid-Level Oral Care Providers

The Virtual Dental Home model in California, USA, provides dental services to vulnerable populations, particularly those who do not receive adequate care in traditional dental settings. By expanding the roles of existing mid-level oral health providers within this model, with an emphasis on prevention and early intervention in oral health processes, preventive services and early interventions in oral health can be effectively implemented [21,22,23].

In Ireland and Australia, mid-level healthcare workers, such as healthcare assistants, play a role in screening and referring patients for further services [24,25,26,27]. In the UK, mid-level practitioners in primary care settings have played a key role in carrying out basic procedures like scaling, preventive restorations, and extractions. Their presence has made it easier to deliver these essential services, ensuring that patients receive prompt and effective care at the initial point of contact [1,28,29].

#### 3.2.2. Dentists

In all the reviewed sources, dentists are identified as central to delivering care. In the United States, dentists are regarded as the leaders of the dental care team and are responsible for formulating treatment plans. The decision-making authority for any restricted treatments by the therapist remains with the dentists [21,30]. In Japan, SCD courses are offered to dentists to prepare them for providing special care services [31].

#### 3.2.3. Special Care Dentists

In Japan, academic internship programs in the field of SCD have been established, and they are conducted under the supervision of the Japan Association for Disability and Oral Health. Dentists are awarded a certificate upon completion of these programs. Additionally, this field is offered as a doctoral program in universities [32]. Australia was among the first countries to officially recognize SCD as a specialty [24]. As a relatively new field, there were only 25 registered specialists in special needs dentistry across Australia in 2024, and in some states and regions, they were either scarce or unavailable [33]. Caregivers in residential and daycare centers commonly reported that the biggest issue was the shortage of dentists with adequate skills in special needs dentistry. Additionally, the lack of dentists willing to treat individuals with disabilities often leads to long waiting lists for these patients [25,34,35].

### 3.3. Care Delivery Model

#### 3.3.1. Virtual Dental Service

The Virtual Dental Home and Apple Tree utilize a cloud-based electronic health record system called DENTICON. This system allows teams to collect information in the field and review it centrally. It enables dentists to access records remotely and enhances team collaboration [21,22,23,36]. In Victoria, Australia, tele-dentistry is an integral part of the dental services offered. Tele-dentistry allows dental professionals to conduct consultations, diagnose conditions, and offer treatment recommendations without the need for in-person visits. This is particularly beneficial for individuals in underserved or remote areas, as well as for those with limited mobility [24,25].

#### 3.3.2. Mobile Dentistry

In the Apple Tree program, mobile and portable equipment is utilized to deliver dental care. Modern “multi-site delivery trucks” specifically designed for this purpose can set up fully equipped mobile dental clinics along a meticulously planned route [36]. The NHS has highlighted mobile dentistry as a component of its services for people with disabilities. However, detailed information about the specific processes and implementation of these mobile dental services is currently limited [37].

#### 3.3.3. Hospital Dental Services

In Victoria and Tasmania, Australia, specialized dental care is provided at the Hospital. This facility offers advanced dental services for complex cases, including orthodontics, periodontics, oral surgery, and prosthodontics [24,25,38].

In Ireland, the primary referral centres for dental services for individuals with disabilities are Dublin Dental University Hospital and Cork University Dental School and Hospital. These facilities provide both secondary and tertiary dental care, along with substantial primary care delivered by dental students [26,27].

In 2012, the Brazilian government policies outlined the provision of oral health services for patients with special needs at the secondary care level or within a hospital system [39,40].

### 3.4. Management

#### 3.4.1. Levels of Healthcare Provision

Service stratification based on the complexity of patient needs is implemented in the United States, the United Kingdom, Brazil, Australia, Canada, and Ireland [24,26,27,41,42]. In Ireland, all special needs schools for children are screened annually, with children then being referred to their local clinic for any necessary treatments. Referrals are made by medical consultants, board-certified dentists, and general dentists from a wide geographic area. In London, all NHS SCD referrals are handled by dentists using a standard proforma and are based on the British Dental Association case-mix model [1,28,29,43]. In Brazil, the majority of individuals with disabilities can receive treatment from general dentists within primary care. Only the more complex cases need to be referred to specialized care [39,40,44,45].

#### 3.4.2. Financial Support

In Japan, national and social health insurance has established premiums for dental treatments for patients with disabilities [31,46,47]. In Germany, a law known as the Versorgungsstrukturgesetz was passed in 2012, marking a significant first step toward implementing the AuB-Konzept. This legislation included “the cost of visits for patients with mobility disabilities” in the health insurance fee scale. While it was a crucial first move to address additional costs, unfortunately, it did not account for expenses related to preventive care [48].

#### 3.4.3. Training

In Canada, advanced courses in Special Care Dentistry (SCD) enable dentists to enhance their knowledge and proficiency in the subject, hence improving their ability to assist individuals with special care needs [42,49]. In Ireland, dental students provide primary care for adults with disabilities as part of their training curriculum. For adults with more complex needs, secondary and tertiary care is provided by dental residents and experienced dentists [26,27]. In Taiwan, dental schools are required to include training in their curriculum on providing care for individuals with disabilities. However, the existing training programs and educational resources for dental students in this area seem to be insufficient [50,51].

## 4. Discussion

The scoping review aimed to identify and categorize the primary and secondary themes involved in the delivery of dental care to PWSNs across various countries. The analysis revealed three primary components: Human Resources, Care delivery model, and Management. Each of these components encompasses several secondary components that contribute to the effectiveness and accessibility of dental care services for PWSNs. This discussion will explore the significance of each primary component and the related subthemes, highlighting the key findings from the reviewed literature.

The delivery of dental care to PWSNs is significantly influenced by a country’s economic status and healthcare policies. High-income nations like the United States, United Kingdom, Australia, and Japan have advanced dental services integrated into universal healthcare systems, allowing for specialized training and innovative care models such as tele-dentistry and mobile units. However, disparities persist, particularly in the U.S., where comprehensive support for PWSNs is limited to a few states like California and Minnesota, which have implemented successful initiatives like the Virtual Dental Home [10,52]. Despite the economic capacity of these countries, barriers such as inadequate training for dental professionals and regional disparities in service availability hinder equitable access to care [53,54]. Furthermore, the lack of standardized training in special needs dentistry exacerbates care disparities globally, highlighting the need for improved educational frameworks and collaborative approaches to meet the unique needs of PWSNs [55].

Middle-income countries like Brazil and Taiwan, despite implementing universal healthcare systems, encounter significant challenges due to economic constraints that lead to uneven access to specialized care and inadequate training resources. In Brazil, the Unified Health System (SUS) aims to provide comprehensive health services, yet it struggles with insufficient public funding and the growing influence of the private sector, which undermines public health initiatives and exacerbates inequalities in access to dental care [56,57,58]. The political landscape also plays a crucial role; Brazil’s democratic system has facilitated health reforms but has been hindered by economic disparities and the expansion of private healthcare, which complicates equitable service delivery [58]. Furthermore, factors such as social, economic, and cultural determinants contribute to disparities in oral health access, highlighting the need for targeted policies to address these inequalities [59]. Thus, the interplay of economic limitations and political structures significantly impacts the effectiveness of healthcare delivery for populations with special needs.

The “Human Resources” component is essential for delivering quality dental care to PWSNs, with three key subthemes: Mid-level Oral Care Providers, Dentists, and SCD. Mid-level practitioners significantly enhance access to dental services, particularly in underserved regions. For instance, California’s Virtual Dental Home model effectively employs mid-level providers to offer preventive care and early interventions, demonstrating a successful integration of these roles into the healthcare system [60]. Similarly, countries like Ireland, Australia, and the UK utilize mid-level healthcare workers for initial screenings and basic procedures, thereby improving service accessibility for PWSNs [61]. However, challenges remain, such as the uneven geographical distribution of dental professionals, which can hinder service delivery [60]. Addressing these disparities through targeted human resources for health (HRH) policies is crucial for ensuring equitable access to quality dental care for all individuals, particularly those with special needs [62].

Dentists play a pivotal role in dental care delivery, particularly for PWSNs. In the United States, they lead dental teams with considerable autonomy, while Japan emphasizes specialized training in SCD to meet the unique needs of PWSNs through dedicated courses [54]. The development of SCD as a recognized specialty in Japan and Australia has led to structured training programs, yet a significant shortage of SCDs persists, particularly in underserved regions [63]. This scarcity results in long waiting times for patients, highlighting the urgent need for increased investment in training and resources for SCDs [55]. Furthermore, the complexity of care required for PWSNs necessitates a patient-centered approach and collaboration among dental professionals to effectively manage their diverse needs [63]. Addressing these challenges is crucial for improving access to quality dental care for this vulnerable population.

The “Care delivery model” theme includes virtual dental home, mobile dentistry, and hospital-based services, all of which are critical in extending care to PWSNs, especially in remote or underserved areas. Research indicates that virtual dental home effectively overcomes geographical barriers, enabling remote consultations and care recommendations through various technologies, including mobile health applications and videoconferencing tools [64,65]. Programs like the Virtual Dental Home and Apple Tree exemplify how cloud-based systems can facilitate collaboration among dental teams, improving patient care delivery [64]. In Australia, virtual dental homes have become integral to extending services in areas with limited dental access, showcasing their role in enhancing care continuity and addressing the needs of underserved populations [65,66]. While a virtual dental home presents significant advantages, it is essential to recognize its limitations, as certain dental procedures still require in-person visits [65].

Mobile dentistry has emerged as a crucial solution for delivering dental care to underserved populations, particularly those with mobility issues or residing in remote areas. Programs like the NHS’s partnership with Dentaid in Hampshire and the Isle of Wight exemplify this approach, providing mobile dental units that cater to individuals facing health inequalities, including families in poverty and those at risk of homelessness [67,68]. In Australia, an interdisciplinary mobile dental service has demonstrated the feasibility of providing tailored care for people with special needs, highlighting the importance of collaboration between private and public sectors to optimize service delivery [69]. While these initiatives underscore the potential of mobile dentistry, further research is needed to evaluate their long-term impact and to refine implementation strategies to ensure comprehensive access for all PWSNs [70].

Hospital-based dental services play a crucial role in managing complex cases, particularly for PWSNs who often require multidisciplinary care. Research indicates that the integration of dental professionals within hospital settings significantly enhances patient outcomes, including a reduction in systemic complications and hospital-acquired infections, which is vital for those with severe conditions [71,72]. The establishment of structured protocols for oral health care in hospitals not only improves the quality of life for hospitalized patients but also optimizes resource use and reduces healthcare costs [73]. Therefore, a comprehensive approach to integrating dental services in hospital environments is essential for addressing the complex dental needs of PWSNs effectively.

The “Management” encompasses Levels of Healthcare Provision and financial support, both of which are essential for ensuring that dental care for PWSNs is adequately organized and funded. Levels of Healthcare Provision based on the complexity of patient needs are a common practice in several countries, including the United States, the United Kingdom, Brazil, Australia, Canada, and Ireland [55,74]. This stratified approach ensures that patients receive care appropriate to their needs, with more complex cases being referred to specialists. In Ireland, for example, special needs schools conduct annual screenings, followed by referrals to local clinics, ensuring that children receive appropriate care in time [74].

Financial support is a critical enabler of dental care services for PWSNs. Various countries have implemented policies to reduce the financial burden on patients, particularly those with disabilities. In Japan, national and social health insurance programs provide coverage for dental treatments for PWSNs, while Germany’s Versorgungsstrukturgesetz includes provisions for covering the costs of visits for patients with mobility disabilities [75]. However, challenges remain, particularly in addressing the costs associated with preventive care, which are not always covered by existing insurance schemes [76]. These financial barriers highlight the need for comprehensive funding mechanisms that support both treatment and preventive care for PWSNs.

Education and training are fundamental to equipping dental professionals with the skills needed to care for PWSNs. Various countries have incorporated special care dentistry into their educational curricula to varying degrees. In Canada, postgraduate training programs and advanced education courses prepare dentists for specialization in SCD. In Ireland, dental students gain hands-on experience in providing primary care for adults with disabilities, while secondary and tertiary care is overseen by more experienced professionals. However, there is a recognized gap in the sufficiency of training programs, as evidenced by Taiwan’s experience, where dental schools are mandated to include training on caring for PWSNs, but existing resources and programs remain inadequate [53,54,77]. This highlights the ongoing need for enhanced educational initiatives to prepare the next generation of dental professionals for the challenges of special care dentistry.

In education, there is an urgent need for interdisciplinary training to equip dental professionals with the skills necessary to address the unique needs of PWSNs, including those with various medical conditions. Developing specialized training programs focused on SCD and promoting continuous education is essential to ensure that dental professionals remain updated on best practices and approaches, ultimately enhancing the quality of care provided to PWSNs.

From a policy perspective, integrating dental care into broader healthcare and social welfare systems is crucial for fostering a holistic approach to care for individuals with special needs. Advocating for financial support and incentives for PWSNs will facilitate their access to essential dental services. Additionally, establishing standards and regulations for SCD practice is vital to ensure consistent quality and patient safety. In clinical practice, implementing a tiered service delivery system tailored to individual complexities and fostering collaboration among dental and healthcare providers will optimize resources and improve overall care coordination, leading to better oral health outcomes for PWSNs.

## 5. Conclusions

In conclusion, the scoping review highlights several crucial components and subthemes essential for the effective delivery of dental care to PWSNs. The findings emphasize the necessity of a well-trained workforce, flexible and accessible service structures, effective management strategies, and comprehensive educational programs. To enhance the quality and accessibility of dental care for PWSNs globally, it is imperative to address the challenges identified in these areas. Each country should align these aspects with its specific context and policy framework to ensure that the unique needs of PWSNs are met effectively and efficiently.

## 6. Limitations

One notable limitation of this scoping review is that the validity and rigor of the studies selected were not critically examined, which may affect the reliability of our findings and the generalizability of the proposed model of care. Additionally, our search did not include regional databases from low- and middle-income countries (LMICs), potentially leading to an underrepresentation of studies from these regions and limiting the comprehensiveness of our insights. The absence of dental-specific databases may also have influenced the scope and quality of studies included, potentially impacting the depth of evidence specific to dental care.

## Figures and Tables

**Table 1:** Primary and Secondary Components for the Strategy of Providing Services to PWSNs.

Primary Components	Secondary Components
Human resources	Mid-level oral care provider
	Dentist
	SCD specialty
Care delivery model	Virtual dental service
	Mobile dentistry
	Hospital dental services
Management	Service categorization
	Financial support
	Training

## Data Availability

Data are contained within the article and Supplementary Materials.

## Supplementary Materials

The following supporting information can be downloaded at: https://www.mdpi.com/article/10.3390/healthcare12232376/s1, Table S1. Search strategy. Figure S1. Selection process for including articles/documents in the study. Table S2. Details of studies included in the scoping review, along with the associated theme [22,23,24,25,26,27,28,29,30,31,34,35,36,38,39,40,41,42,44,45,48,49,50,78,79,80,81,82,83,84,85,86,87,88,89,90,91,92].
