# Supplementary material for: Accessibility of Special Care Dentistry Across Countries: A Scoping Review
Source: Healthcare (Basel). 2024 Nov 26;12(23):2376. doi: 10.3390/healthcare12232376 (PMC11641257; doi:10.3390/healthcare12232376)
Supplement: Supplementary file 1 [file healthcare-12-02376-s001.zip › healthcare-3243329-supplementary.pdf]

Table S1. Search strategy.

Database	Queries
PubMed	("Disabled Persons"[MeSH] OR "Handicapped"[MeSH] OR "Down Syndrome"[MeSH] OR "Communication Disorders"[MeSH] OR "Intellectual Disability"[MeSH] OR "Aged"[MeSH] OR "Persons with Special Health Care Needs"[MeSH]) AND ("Oral Health"[MeSH] OR "Dentistry"[MeSH] OR "Dental Health Services"[MeSH]) AND ("Health Services Accessibility"[MeSH] OR "Health Care Utilization"[MeSH] OR "Patient Acceptance of Health Care"[MeSH] OR "Delivery of Health Care"[MeSH] OR "Access to Health Care"[MeSH])
Scopus	(disability * OR disabled OR handicap * "down syndrome" OR (communicat * AND (disorder OR dysfunction) OR retard * OR (mental AND deficien*) OR old * OR elder * OR "Special care need*") AND (dent* OR oral) AND (service OR care OR system OR delivery)
Embase	1. disabilit*.mp.<br>2. disabled.mp. or *disabled person/<br>3. handicap*.mp.<br>4. *Down syndrome/<br>5. *communication disorder/<br>6. mental retard.mp. or *mental deficiency/<br>7. mental deficiency/ or mental deficien.mp.<br>8. Special care need.mp.<br>9. *dentistry/ or dentistry.mp.<br>10. oral care.mp.<br>11. special care dentistry.mp.<br>12. 9 or 10 or 11<br>13. health service/ or service.mp.<br>14. health care/ or care.mp.<br>15. delivery.mp.<br>16. 13 or 14 or 15<br>17. 1 or 2 or 3 or 4 or 5 or 6 or 7 or 8<br>18. 12 and 16 and 17

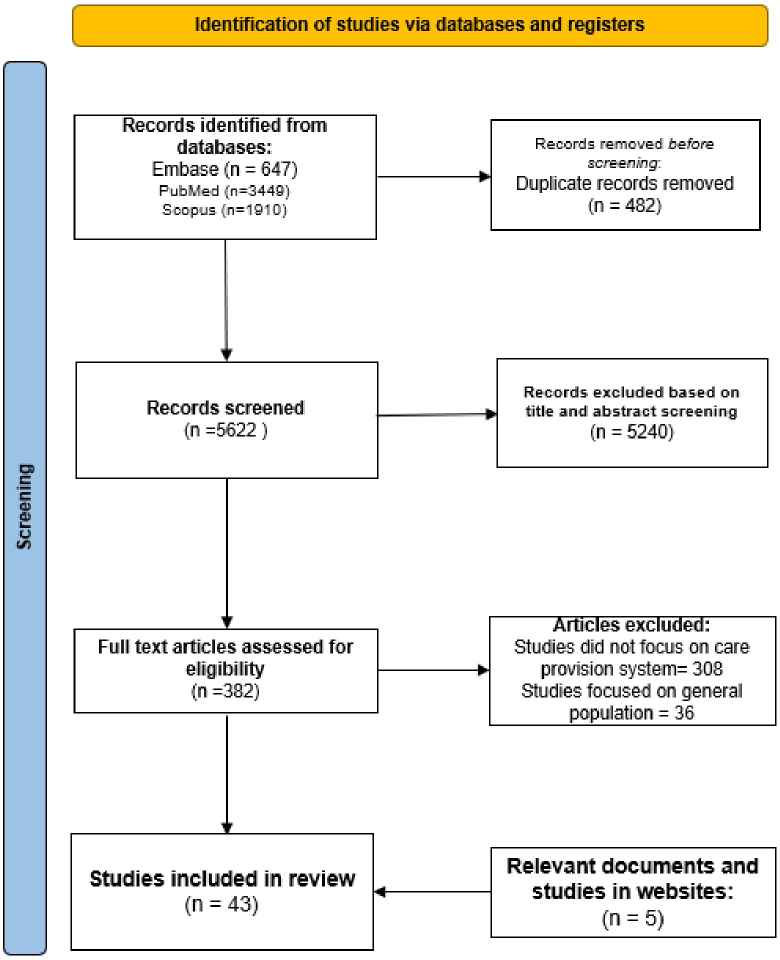

Figure S1. Selection process for including articles/documents in the study.

Table S2. Details of studies included in the scoping review, along with the associated theme.

	Author	Year	Country	Source Type	Themes		
					**Human Resources**	**Care Delivery Model**	**Management**
1.	Faulks et al. [32]	2012	Japan	Article	✓	✓	✓
2.	Tokunaga et al. [46]	2015	Japan	Article	✓	✓	-
3.	The Japanese Society for Disability and Oral Health [78]	2024	Japan	Website	✓	-	-
4.	Ishimaru et al. [47]	2019	Japan	Article	✓	-	-
5.	Glassman et al. [79]	2008	USA	Article	✓	✓	✓
6.	Seirawan et al. [80]	2008	USA	Article	-	✓	-
7.	Glassman et al. [21]	2012	USA	Article	✓	✓	✓
8.	Advancing oral health in America [81]	2011	USA	Book	-	✓	-

9.	Improving access to oral health care for vulnerable and underserved populations. [22]	2012	USA	Book	-	✓	✓
10.	Glassman et al. [23]	2012	USA	Article	-	✓	✓
11.	Jacobi et al. [36]	2015	USA	Article	✓	-	-
12.	Helgeson et al. [82]	2013	USA	Article	✓	-	-
13.	Glassman et al. [30]	2014	USA	Article	-	✓	✓
14.	Glassman et al. [83]	2016	USA	Article	-	✓	✓
15.	Cruz et al. [84]	2016	USA	Article	✓	-	-
16.	Condessa et al.[39]	2020	USA	Article	✓	-	-
17.	Junqueira et al. [40]	2008	USA	Article	✓	-	-
18.	Gavina et al. [44]	2019	USA	Article	✓	-	-
19.	Pedrazzi et al. [45]	2008	USA	Article	✓	-	-
20.	Mugayar et al. [85]	2007	USA	Article	✓	-	-
21.	Pucca et al. [41]	2015	USA	Article	✓	-	-
22.	Lim et al. [24]	2020	Australia	Article	-	✓	-
23.	Mariño et al. [25]	2014	Australia	Article	✓	-	✓
24.	New South Wales Government. [86]	2024	Australia	Website	-	✓	-
25.	Scrine et al. [34]	2019	Australia	Article	✓	-	-
26.	Webb et al. [35]	2013	Australia	Article	-	-	✓
27.	Tasmania Government Special care dental health services. [87]	2018	Australia	Document	-	✓	-
28.	Lim et al. [38]	2017	Australia	Article	✓	✓	-
29.	Salmasi et al. [51]	2015	Canada	Article	✓	✓	✓
30.	Improving access to oral health care for vulnerable people living in Canada. [44]	2014	Canada	Document	✓	✓	✓
31.	Ziller et al. [50]	2015	Germany	Article	-	-	✓
32.	Phadraig et al. [27]	2017	Irland	Article	✓	✓	✓
33.	AlKindi et al. [28]	2016	Irland	Article	✓	✓	✓
34.	Kravitz et al. [88]	2015	Sweden	Article	✓	✓	✓
35.	Pälvärinne et al. [89]	2018	Sweden	Article	-	✓	✓
36.	Niiranen et al. [90]	2008	Finland	Article	✓	-	✓
37.	Jeng et al. [50]	2009	Taiwan	Article	✓	-	-
38.	Yu et al. [51]	2022	Taiwan	Article	✓	-	-
39.	Griffiths et al. [28]	2016	UK	Article	✓	✓	✓

40.	Guides for commissioning dental specialties–Special Care Dentistry. [91]	2015	UK	Document	-	✓	-
41.	Gallagher et al. [1]	2007	UK	Article	-	✓	✓
42.	Baird et al. [29]	2008	UK	Article	✓	✓	✓
43.	Dyer et al. [92]	2023	UK	Article	✓	-	✓
